# Effect of sevoflurane post-conditioning on postoperative cognitive function in carotid endarterectomy patients: a randomized, double-blind, controlled trial

**DOI:** 10.3389/fmed.2025.1716370

**Published:** 2025-12-08

**Authors:** Bin Wang, Shuangjiang Li, Chaoqiong Wang, Jiali Zhu, Ying Jin, Dawei Sun, Junhui Lang

**Affiliations:** 1 Department of Anesthesiology, The Second Affiliated Hospital, Zhejiang University School of Medicine, Hangzhou, China; 2 Zhejiang Key Laboratory of Pain Perception and Neuromodulation, Hangzhou, China; 3 Department of Rehabilitation in Traditional Chinese Medicine, The Second Affiliated Hospital of Zhejiang University School of Medicine, Hangzhou, Zhejiang, China; 4 Center for Rehabilitation Medicine, Department of Anesthesiology, Zhejiang Provincial People’s Hospital (Affiliated People’s Hospital, Hangzhou Medical College), Hangzhou, Zhejiang, China; 5 Research Institute of Anesthesiology and Perioperative Medicine, Hangzhou Medical College, Hangzhou, Zhejiang, China

**Keywords:** cognition, sevoflurane, carotid endarterectomy, neurocognitive recovery, postoperative cognition, anesthesia effects

## Abstract

**Purpose:**

This study evaluated the effect of low-dose sevoflurane post-conditioning on early neurocognitive recovery in patients aged 50–80 years undergoing carotid endarterectomy (CEA).

**Methods:**

In this randomized, double-blind, single-center trial, 71 patients undergoing elective CEA receiving either sevoflurane post-conditioning (Group S, *n* = 36) or standard propofol-based anesthesia (Group P, *n* = 35) were analysed. Cognitive function was assessed using the Mini-Mental State Examination (MMSE) and Montreal Cognitive Assessment (MoCA) at baseline, and on postoperative days 1 and 3. The primary outcome was the change in cognitive scores from baseline.

**Results:**

The two-way repeated-measures ANOVA revealed a significant main effect of group for both MMSE (*p* < 0.001) and MoCA (*p* = 0.019) scores. As the groups were comparable at baseline, this difference emerged postoperatively, driven by divergent outcomes on day 1: the propofol group (Group P) experienced a significant change in cognitive scores from baseline in both MMSE (−0.97 ± 1.81) and MoCA (−0.80 ± 1.83), whereas the scores in the sevoflurane group (Group S) were preserved in both MMSE (+0.58 ± 1.76) and MoCA (+0.81 ± 2.59). A significant between-group difference was therefore evident on postoperative day 1 (MMSE *p* = 0.002; MoCA *p* = 0.037) and was maintained on postoperative day 3 (MMSE *p* = 0.023; MoCA *p* = 0.036).

**Conclusion:**

Low-dose sevoflurane post-conditioning provides superior short-term preservation of cognitive function compared to standard anesthesia in patients undergoing CEA. These findings support the potential neuroprotective role of sevoflurane post-conditioning. Further research is warranted to explore long-term outcomes that incorporate biological sample testing.

**Clinical trial registration:**

https://ClinicalTrials.gov, NCT04950205.

## 1 Introduction

Perioperative neurocognitive disorders (NCDs), such as delirium and postoperative cognitive dysfunction (POCD), are the predominant postoperative complications in older patients (1). The incidence of postoperative delirium ranges from 11 to 51%, depending on the type of procedure, while POCD incidence varies between 9.9 and 40% (2, 3). Delayed neurocognitive recovery or POCD significantly impacts long-term outcomes, manifesting as early withdrawal from the workforce, increased reliance on social welfare, and heightened mortality rates (4).

Carotid endarterectomy (CEA) is the primary intervention for treating severe carotid artery stenosis and preventing ischemic stroke. However, 19% patients undergoing CEA experience cognitive dysfunction, potentially resulting from intraoperative vascular forceps clamping or plaque dissection can lead to the rupture of unstable plaques, releasing cholesterol crystals, fibrous cap fragments and calcified granules. When the blood vessels are opened during the operation, air enters the circulation, and tiny bubbles block the cortical arterioles. After carotid artery occlusion during the operation, due to insufficient compensation of the contralateral carotid artery or lateral branches of the posterior circulation, it can lead to a decrease in cerebral blood flow and result in insufficient cerebral blood supply. The sudden increase in cerebral blood flow in the long-term hypoperfusion area after revascularization causes ischemia–reperfusion injury, hyperperfusion syndrome and oxidative stress (5–7).

Propofol, the most widely used intravenous anesthetic drug currently, can inhibit the damage of oxygen free radicals in ischemic brain circulation, which has a protective effect on cerebral ischemia reperfusion injury and can improve the cognitive function after CEA surgery (8). Despite these promising preclinical characteristics, however, the incidence of postoperative cognitive dysfunction (POCD) remains high in patients receiving propofol-based anesthesia (9). This persistent clinical challenge highlights a critical gap in current neuroprotective strategies and justifies the investigation of alternative agents, such as sevoflurane, which may offer a more effective approach.

Sevoflurane is a common drug used for maintenance under inhalation general anesthesia, which is easy to use and highly controllable. Low concentration of sevoflurane can reduce brain tissue metabolism and play a brain protective role (10). Prior research by our team demonstrated that sevoflurane post-conditioning mitigates myocardial infarction size and apoptosis in myocardial ischemia–reperfusion injury models, suggesting its potential applicability in cerebral protection (11). Although some studies have suggested that sevoflurane exposed elderly rats would show postoperative cognitive dysfunction with the increase of sevoflurane concentration (4, 12). Sevoflurane preconditioning or post-conditioning showed significant improvement in ischemia reperfusion injury and cognitive dysfunction outcomes (13, 14). Sevoflurane preconditioning has been extensively validated for mitigating ischemia–reperfusion injury (15, 16). Studies have shown that activating the classical Notch signaling pathway may be involved in the neuroprotective effect induced by sevoflurane preconditioning. In the rat experiment, sevoflurane pretreatment increased the levels of Notch intracellular domain (NICD) protein and Hes messenger RNA in the ischemic penumbra brain tissue, exerting a neuroprotective effect (17). However, sevoflurane post-conditioning is easier to achieve in clinical work, and post-conditioning has been proved to reduce nerve apoptosis and play a neuroprotective role in both *in vitro* and animal models (18, 19). Based on this evidence, we hypothesize that sevoflurane post-conditioning could similarly protect against cerebral ischemia–reperfusion injury in human patients.

This study investigated whether low concentration of sevoflurane has a protective effect on the brain after ischemia in patients undergoing carotid artery dissection; moreover, the incidence of adverse events was also observed. We hypothesized that patients undergoing carotid endarterectomy (CEA) who receive post-conditioning with 1% sevoflurane would demonstrate significantly improved early delayed neurocognitive recovery, as measured by higher postoperative Mini-Mental State Examination (MMSE) and Montreal Cognitive Assessment (MoCA) scores, compared to patients receiving propofol-based anesthesia alone.

## 2 Methods

### 2.1 Study design and population

This single-center, randomized, double-blinded, clinical trial was conducted from July 1, 2021, to December 31, 2022. A consistent medical team oversaw all patient care. The study adhered to the ethical principles outlined in the Declaration of Helsinki and received approval from the hospital’s Ethics Review Committee (Approval No. (2021) 0449). Written informed consent was obtained from all participants before enrollment. The trial was registered on ClinicalTrials.gov (NCT04950205), detailing its design and objectives. The study also complied with the Consolidated Standards of Reporting Trials (CONSORT) guidelines (20).

### 2.2 Participants

Participants were assessed one day prior to surgery. Inclusion criteria were: (1) undergoing unilateral CEA surgery, (2) aged 50–80 years, and (3) planned elective general anesthesia. Exclusion criteria included: (1) preoperative cognitive dysfunction or dementia, (2) history of prior CEA surgery, (3) preoperative MMSE score < 20, (4) illiteracy, (5) inability to cooperate due to mental illness, (6) participation in other clinical trials, (7) neuroelectrophysiological monitoring abnormalities or unexpected events such as massive intraoperative bleeding, and (8) postoperative ICU admission.

### 2.3 Randomization and masking

Randomization was conducted using a simple randomization method, with the randomization sequence generated by a statistician who was not involved in the clinical procedures. Each participant was assigned a unique random number. The randomization sequence was implemented using a sealed envelope system. Before surgery, the anesthesiologist opened the envelope to reveal the group allocation and carried out the corresponding anesthetic protocol.

Participants were randomly assigned to either the intraoperative propofol intravenous anesthesia group (Group P, *n* = 35) or the 1% sevoflurane post-conditioning group (Group S, *n* = 36). For Group S, 1% sevoflurane inhalation began immediately after carotid artery opening on the operative side, with propofol concentrations adjusted as necessary. Anesthesia depth was maintained with BIS values between 40 and 60, and blood pressure was managed according to the surgical procedure.

To ensure masking, the anesthesiologist performing the surgery was aware of the group allocation, but the cognitive function assessments were conducted by independent evaluators who were blinded to the group assignments. This double-blind design minimized potential biases during outcome assessment. Cognitive function was evaluated on the first day before surgery, the first day after surgery, and the third day after surgery.

### 2.4 Anesthesia and intraoperative management

Patient were instructed to fast for 8 h before surgery. Upon entering the operating room, internal jugular vein and radial artery catheterization were performed under ultrasound guidance and continuous blood pressure was monitored. Routine monitoring included heart rate (HR), electrocardiogram (ECG), oxygen saturation (SpO2), noninvasive blood pressure (NIBP), and end-tidal carbon dioxide (PETCO2) for general physiological assessment. Intraoperative brain function on the affected side was specifically monitored using bispectral index (BIS), somatosensory evoked potential (SEP), and motor evoked potential (MEP).

Induction of anesthesia utilized sufentanil (0.5 μg/kg), etomidate (0.2 mg/kg), and cis-atracurium (0.2 mg/kg), with tracheal intubation performed via video laryngoscopy to enable artificial ventilation. Anesthesia maintenance was achieved using intermittent positive pressure ventilation (IPPV), with tidal volumes of 8–10 mL/kg, a respiratory rate of 10–12 breaths per minute, and PETCO2 controlled within 35–40 mmHg. Neuroelectrophysiological monitoring (SEP and MEP) was utilized throughout the surgery, with abnormalities (e.g., a 50% reduction in wave amplitude or a 10% latency extension) leading to exclusion from the study.

Participants were randomly assigned to either the propofol-based intravenous anesthesia group (Group P, *n* = 35) or the propofol-based anesthesia with 1% sevoflurane post-conditioning group (Group S, *n* = 36). For Group P, anesthesia was induced and maintained using propofol via intravenous infusion, with dosages adjusted to maintain appropriate anesthesia depth. For Group S, anesthesia was similarly induced and maintained with propofol, but 1% sevoflurane inhalation was initiated immediately after carotid artery opening on the operative side, with propofol concentrations adjusted as necessary to balance the combined effect. In both groups, anesthesia depth was maintained with Bispectral Index (BIS) values between 40 and 60, and blood pressure was managed according to the surgical procedure. According to expert guidelines for carotid endarterectomy (CEA) surgery, during carotid artery clamping, mean arterial pressure (MAP) was increased by 10–20% using deoxyadrenalin and reduced by 10–20% following arterial opening (21, 22).

The choice of 1% sevoflurane as the post-conditioning dose was based on several considerations. The minimal alveolar concentration (MAC) of sevoflurane is 1.71%, defined as the alveolar concentration at which 50% of patients do not respond to surgical skin incision under one atmosphere of pressure, indicating that 1% sevoflurane represents a low dose during anesthesia maintenance. Previous clinical studies have demonstrated that low-dose sevoflurane provides cardioprotective effects during CEA surgery. The 1% concentration was selected as it offers cardioprotection while minimizing potential adverse effects associated with higher dose (8). Additionally, this concentration is easy to control and monitor in clinical practice, facilitating standardized implementation in research settings.

### 2.5 Outcomes

The primary outcome was the decline in cognitive function on the first postoperative day, as measured by the differences in Mini-Mental State Examination (MMSE) and Montreal Cognitive Assessment (MoCA) scores compared to the preoperative baseline.

Secondary outcomes included physiological parameters such as heart rate, invasive arterial blood pressure, BIS, and PETCO2, which were recorded at three time points: 5 min before anesthesia induction (T1), immediately after carotid artery occlusion (T2), and 10 min after carotid artery opening (T3). Sedation-agitation levels were evaluated using the Ricker Sedation-Agitation Scale (SAS) 5 min after extubation in the postanesthesia care unit (PACU). Postoperative adverse events, including cerebral infarction and cerebral perfusion syndrome, were also recorded.

## 3 Statistical analysis

### 3.1 Sample size calculation

In the pilot study, MMSE scores decreased by 2.1 in Group P and 1.1 in Group S on the first postoprative day, with a standard deviation (SD) of 1.50. Based on a power of 0.8 and an alpha level of 0.05, PASS software (NCSS, LLC, Kaysville, Utah, United States) determined a required sample size of 37 participants per group. Accounting for a 10% loss to follow-up, 42 participants per group were required. For MoCA, the scores declined by 1.8 in Group P and 0.5 in Group S, with an SD of 1.82. Using the same parameters, the required sample size was 32 participants per group, which increased to 36 after accounting for a 10% loss to follow-up. Considering both scores, the study opted for 42 participants per group, totaling 84 participants, randomized in a 1:1 ratio.

### 3.2 Outcome analysis

Statistical analysis was performed using SPSS 26.0 software (IBM, United States). A *p*-value < 0.05 was considered statistically significant. First, baseline demographic and clinical characteristics were compared between the two groups. For normally distributed continuous data (e.g., age), the independent-samples *t*-test was used. For non-normally distributed data, the Mann–Whitney U test was applied. Categorical data (e.g., gender) were compared using the Pearson chi-square test.

To assess the primary outcomes, the changes in MMSE and MoCA scores over time were analyzed using a two-way repeated-measures analysis of variance (ANOVA). In this model, “group” (Group S vs. Group P) was included as the between-subjects factor, and “time” (preoperative, postoperative day 1, and postoperative day 3) was the within-subjects factor. Our analysis first examined the time × group interaction effect. If the interaction was significant, simple effects tests would be conducted. If the interaction was not significant, as was the case in our study, we proceeded to interpret the main effects of group and time. *Post-hoc* comparisons with Bonferroni correction were then used to identify specific between-group differences at each time point and within-group changes from baseline where appropriate.

## 4 Results

### 4.1 Patients and baseline characteristics

**Figure 1:**
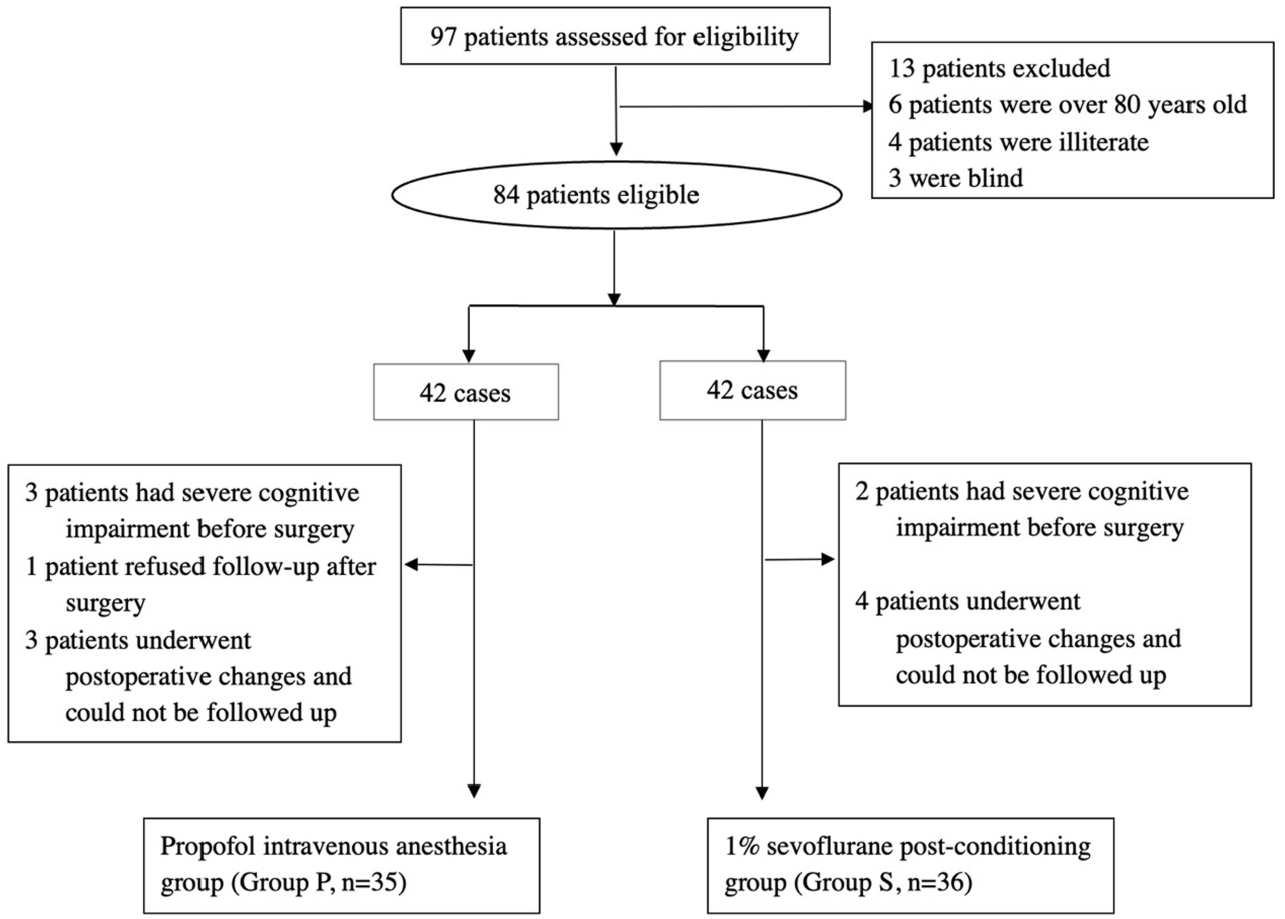
Patients’ recruitment and follow-up flow chart.

A total of 97 patients were screened for eligibility, with 26 excluded from the study. The reasons for exclusion were as follows: 6 patients were over 80 years old, 4 patients were illiterate, and 3 patients were blind. A total of 84 patients were included in the study. However, 5 patients had severe cognitive impairment before surgery, 1 patient refused follow-up after surgery, and 7 patients were excluded due to postoperative complications, including cerebral hemorrhage, cerebral infarction, and transfer to the ICU (Figure 1).

The final analysis included 71 patients, with 35 patients in the propofol intravenous anesthesia group (Group P) and 36 in the 1% sevoflurane post-conditioning group (Group S). Baseline characteristics showed no significant differences between the groups (Table 1).

**Table 1:** Baseline characteristics.

Characteristics	Group P (*n* = 35)	Group S (*n* = 36)	*P*-value
Age (yr)	70.1 ± 7.1	66.9 ± 8.8	0.246
Height (m)	1.66 ± 0.05	1.66 ± 0.07	0.800
Weight (kg)	63.8 ± 8.5	65.1 ± 10.4	0.285
BMI (kg m^−2^)	23.3 ± 3.1	23.7 ± 3.4	0.572
Male gender	31	29	0.545
Education (yr)	8.3 ± 2.8	8.2 ± 2.9	0.182
ASA physical status			
2	17	18	0.904
3	18	18	1
SAS score	4.00 ± 0.59	4.03 ± 0.65	0.44
Hypertension	16 (45.7%)	22 (61.1%)	0.193
Diabetes	9 (25.7%)	10 (27.2%)	0.844
Hyperlipidemia	19 (54.3%)	18 (50.0%)	0.718
ACEI/ARB	9 (25.7%)	11 (30.6%)	0.650
Calcium antagonists	10 (25.7%)	11 (30.6%)	0.855
Hypoglycemic drugs	9 (25.7%)	10 (27.2%)	0.844
Antiplatelet drugs	16 (45.7%)	17 (47.2%)	0.899
Statins	21 (60.0%)	18 (50%)	0.397

Data are presented as mean (SD), median (interquartile range) or number of patients (%).

ASA, American Society of Anesthesiologists; BMI, Body Mass Index; SAS, Score Sedationagitation Scale; ACEI, Angiotensin-Converting Enzyme Inhibitor, ARB, Angiotensin II Receptor Blockers.

### 4.2 Perioperative hemodynamics

**Figure 2:**
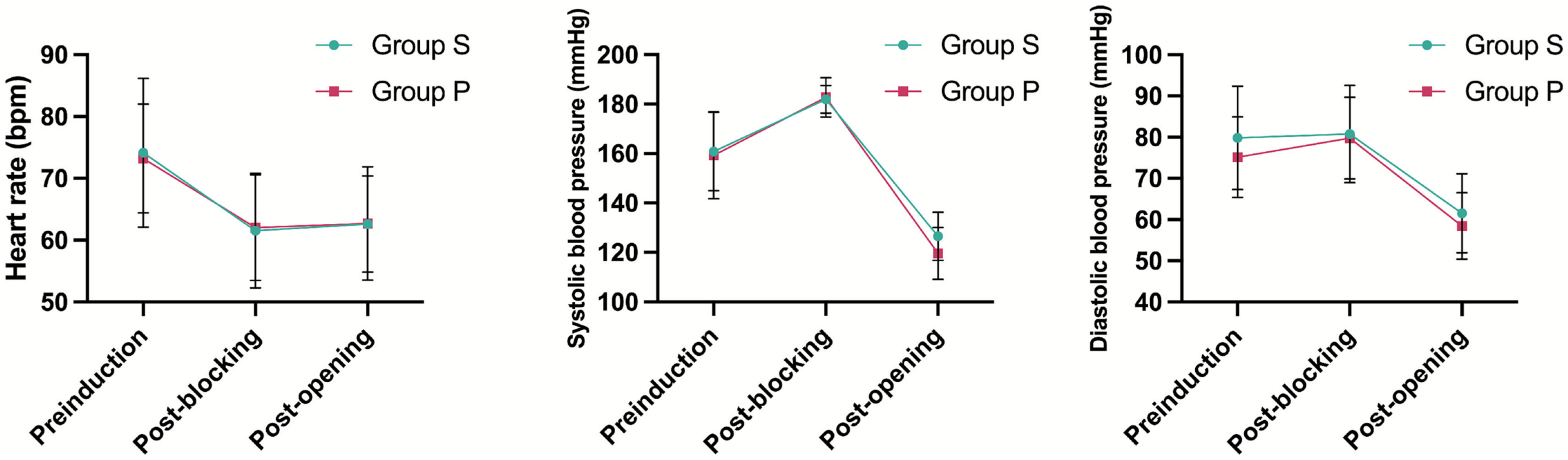
Patient’s vital signs changed during operation.

There were no significant differences in any of the hemodynamic parameters between Groups P and S (Figure 2).

### 4.3 Cognitive outcomes

Preoperatively, there were no significant differences between groups in either the MMSE or the MoCA scores (*p* = 0.547 and *p* = 0.943, respectively), establishing a comparable baseline. The primary analysis using a two-way repeated-measures ANOVA revealed a significant main effect of group for both MMSE (*p* < 0.001) and MoCA (*p* = 0.019). However, the time × group interaction effect was not significant for either measure (MMSE: *p* = 0.141; MoCA: *p* = 0.198).

The significant overall difference between the groups was driven by divergent outcomes on postoperative day 1. At this time point, the Group P experienced a significant decline from baseline in both MMSE (mean change, −0.97 ± 1.81; *p* = 0.003) and MoCA (mean change, −0.80 ± 1.83; *p* = 0.014). In contrast, cognitive function in the Group S was preserved, showing a slight increasement in both MMSE (mean change, +0.58 ± 1.76) and MoCA (mean change, +0.81 ± 2.59). Consequently, Group S had significantly higher absolute scores than Group P on day 1 (MMSE: *p* = 0.002; MoCA: *p* = 0.037).

**Figure 3:**
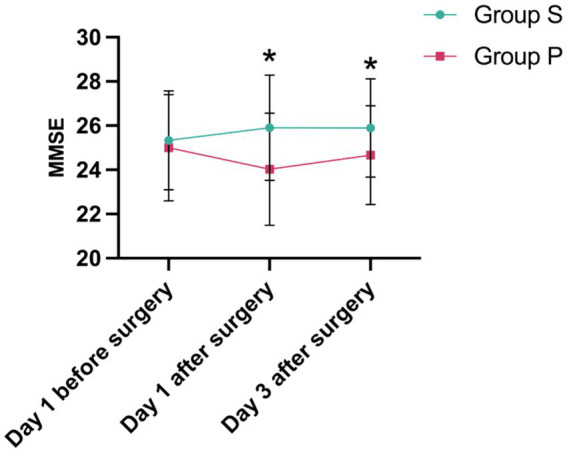
Mini-Mental State Examination (MMSE). * indicates statistical significance (*p* < 0.05).

**Figure 4:**
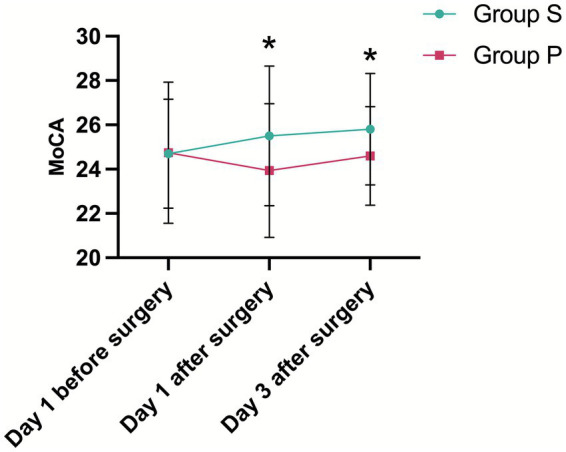
Montreal Cognitive Assessment (MoCA). * indicates statistical significance (*p* < 0.05).

This between-group difference was maintained through postoperative day 3 (MMSE: *p* = 0.023; MoCA: *p* = 0.036). By this time, while Group P showed signs of recovery from their day 1 nadir, their cognitive scores remained below their preoperative baseline (MMSE change from baseline, −0.34 ± 1.51; MoCA change from baseline, −0.14 ± 1.73). Conversely, Group S maintained a cognitive performance level consistently above their baseline (MMSE change from baseline, +0.56 ± 1.84; MoCA change from baseline, +1.11 ± 2.23). In summary, sevoflurane post-conditioning was associated with a superior preservation of early postoperative cognitive function, as evidenced by significantly higher MMSE and MoCA scores on both the first and third postoperative days (Figures 3, 4).

## 5 Discussion

This randomized controlled trial demonstrated that low-dose sevoflurane post-conditioning significantly attenuated early postoperative cognitive decline in patients aged 50–80 years undergoing carotid endarterectomy. Specifically, patients receiving sevoflurane post-conditioning (Group S) maintained significantly higher MMSE and MoCA scores on both postoperative day 1 and day 3 compared to the propofol group. These findings support our hypothesis that sevoflurane post-conditioning can serve as a neuroprotective strategy against ischemia–reperfusion injury during CEA, offering a promising approach to preserve short-term cognitive function in this vulnerable patient population.

Sevoflurane post-conditioning can reduce the levels of blood lipids and malondialdehyde in rats with cerebral ischemia–reperfusion, increase the density of normal pyramidal neurons, and counteract neurotoxicity caused by oxidative stress. It can also up-regulate the expression of NQO1 in ischemic brain tissue by activating the PI3-K/AKT pathway, exerting a cerebral protective effect. It works through mechanisms such as regulating cerebral blood flow, inhibiting the release of inflammatory factors, and reducing neuronal apoptosis (23). We must emphasize, however, that these proposed mechanisms are largely extrapolated from animal models. Their direct applicability to patients undergoing carotid endarterectomy remains speculative, and our clinical trial was not designed to investigate these underlying pathways. Our findings robustly demonstrate a clinical neuroprotective effect; however, the precise mechanistic basis for this effect in humans warrants further dedicated investigation.

Previous research in humans on the effects of sevoflurane vs. propofol on postoperative cognition has yielded conflicting outcomes. Older adults are particularly vulnerable to cognitive dysfunction after major surgery (3, 24, 25). A multicenter randomized trial in China involving 1,228 elderly patients undergoing major tumor surgery reported a roughly one-third reduction in postoperative delirium incidence in the propofol group compared to the sevoflurane group, with the difference primarily observed on the first postoperative day (26). Conversely, a recent multicenter randomized controlled study examined the impact of propofol and sevoflurane on delayed postoperative cognitive recovery in valve replacement surgery patients. The incidence of cognitive impairment 7–14 days post-surgery was significantly lower in the sevoflurane group (31.9%) compared to the propofol group (43.4%) (27). The reasons for this contradictory conclusion are not clear. These contradictory findings may stem from differences in patient characteristics, including age ranges and types of surgeries. Taking all the evidence together, the role of anesthetics in delayed neurocognitive recovery remains controversial remains controversial (28–31).

Compared to previous trials, our study had notable distinctions. First, our study focused on patients undergoing CEA, a population at high risk for cognitive impairment. Second, we specifically examined the short-term effects of low-dose sevoflurane. Third, our intervention targeted the post-ischemia reperfusion period, a critical time point for mitigating ischemia–reperfusion injury.

### 5.1 Limitations

This study has several limitations that should be acknowledged. Firstly, the single-center design and the deliberate selection of patients undergoing CEA by the same surgeon, while intended to minimize surgical variability, inherently restricted our sample size. This smaller cohort may limit the statistical power to detect more subtle intervention effects and could affect the generalizability of our findings to broader populations or diverse surgical teams. Future multi-center studies with larger sample sizes are warranted to validate these results.

Secondly, we did not collect perioperative blood or cerebrospinal fluid biomarkers. Although there are no specific biomarkers for cognitive function currently, cortisol can be used as an important indicator of perioperative stress in the aged under different anesthesia methods (32). However, in the long-term follow-up study of elderly patients with propofol and inhalation anesthesia, Villalobos et al. screened cerebrospinal fluid for biomarkers associated with Alzheimer’s disease, such as Ab42 and tau, which was beyond the scope of our study (33).

Thirdly, our study was characterized by a limited duration of follow-up, and we did not collect long-term cognitive outcome data. The decision to curtail long-term follow-up was due to anticipated difficulties in reliably administering the chosen cognitive assessments without face-to-face interaction, which we believed could compromise data quality. Consequently, the persistence of the observed cognitive effects beyond our follow-up period remains undetermined.

Fourthly, despite employing mitigation strategies such as using alternate test versions and restricting test duration, the potential for practice effects from repeated cognitive assessments cannot be entirely excluded. While a standardized evaluation process was maintained for all participants, repeated exposure to assessment tools may have influenced performance over time (34).

Fifthly, while the between-group differences in cognitive scores were statistically significant, the absolute magnitude of these differences did not meet the traditional thresholds for a Minimal Clinically Important Difference (MCID) often cited for neurodegenerative diseases. However, we contend that the concept of MCID is highly context-dependent. In the acute perioperative setting of our study, the primary therapeutic goal was the prevention of cognitive decline, a distinct and negative neurological event. Therefore, we interpret the statistically significant prevention of the decline seen in the propofol group as a clinically meaningful outcome, even in the absence of a large score improvement. Future research could incorporate more sensitive neuropsychological batteries or patient-reported outcomes to further explore the real-world impact of preventing such subtle cognitive insults.

## 6 Conclusion

This study demonstrates that sevoflurane post-conditioning provides a significant short-term neuroprotective benefit, improving early neurocognitive recovery as assessed by MMSE and MoCA scores on postoperative days 1 and 3 following carotid endarterectomy, suggesting a clinically relevant neuroprotective anesthetic approach. These promising findings highlight the potential benefits of this intervention; however, to further substantiate these results, elucidate the long-term durability of the observed cognitive improvements, and explore the underlying mechanisms, future investigations should prioritize larger, multicenter randomized controlled trials that incorporate biological sample testing.

## Data Availability

The original contributions presented in the study are included in the article. Further inquiries can be directed to the corresponding authors, and raw data may be made available upon reasonable request, subject to patient privacy protection.
